# Intravenous Immunoglobulin-Associated Elevation of Liver Enzymes in Neurological Autoimmune Disorder: A Case Series

**DOI:** 10.7759/cureus.10020

**Published:** 2020-08-25

**Authors:** Anupama Behera, Satyabrata Guru

**Affiliations:** 1 Internal Medicine, All India Institute of Medical Sciences, Bhubaneswar, IND; 2 Trauma and Emergency/Internal Medicine, All India Institute of Medical Sciences, Bhubaneswar, IND

**Keywords:** transaminitis, intravenous immunoglobulin, neurological autoimmune disorders

## Abstract

Intravenous immunoglobulin (IVIG) is used in the treatment of a variety of autoimmune neurological disorders and is generally regarded as safe. We present a case series where IVIG causes transaminitis. The adverse effects are mostly due to the stabilizing agent used to prepare the IVIG (e.g., maltose in this series). While the adverse effects are usually self-limiting, physicians should be cautious in administering an IVIG preparation to these patients.

## Introduction

Intravenous immunoglobulin (IVIG) is used as an immunomodulatory agent in many neurological autoimmune disorders, such as Guillain-Barré (GBS) syndrome, chronic inflammatory demyelinating polyneuropathy, multifocal motor neuropathy, inclusion body myositis, optic neuropathy of unknown origin, myasthenia gravis, and multiple sclerosis. We reported prospectively on the adverse effects of IVIG in a case series of 11 patients, including 10 cases of GBS and one case of myasthenic crisis. The prevalence of IVIG-related complications has changed in clinical practice due to its widespread use. Usually, IVIG is safe; however, a retrospective study showed a high rate of complications of up to 81% [1,2]. These included common adverse effects, such as allergic reaction, headache, nausea, fatigue, chills, fever, thrombosis, and elevation of liver enzymes. Here, we studied the adverse effects of IVIG, especially transaminitis, under clinical conditions including GBS and myasthenia gravis.

## Case presentation

GBS cases

We reported 10 cases of GBS in men and women of different ages who presented with a history of acute onset symmetrical, ascending, areflexic paraplegia/quadriplegia and an antecedent history of upper respiratory tract/gastrointestinal tract infection. On examination, there was bilateral facial nerve palsy, absent deep tendon reflexes, proximal weakness more than distal weakness, and bilateral decreased vibration and position sensation without bladder bowel involvement. All hematological parameters and renal function test were within normal limit. Most of the patients developed transaminitis without jaundice during course of their treatment that was self-limiting. Cerebrospinal fluid analysis showed an elevated protein level with a normal cell count. Nerve conduction studies revealed acute inflammatory demyelinating polyneuropathy, acute motor axonal neuropathy, and acute motor-sensory axonal neuropathy. All patients were treated with IVIG for five days for a total dose of 2 gm/kg body weight. All patients were discharged in stable condition within three to four weeks of admission.

Myasthenic crisis case

A 45-year-old man presented with bilateral ptosis, diplopia, and rapidly progressive proximal limb weakness associated with respiratory distress. He was clinically and electrophysiologically proven along with positive antibodies and diagnosed with myasthenia gravis. All hematological parameters and renal function test were within normal limit. He developed transaminitis without jaundice during course of his treatment that was self limiting. The patient was managed with a mechanical ventilator and IVIG in a dose of 0.4 gm/kg body weight daily for five days and was discharged in stable condition after a two-week hospital stay. 

All patients were admitted during the study period of January 2018 to December 2019. We prospectively studied the side effects of IVIG during the treatment of eleven patients, of whom nine patients (81.81%) demonstrated significant elevation of liver enzymes within two to three days following IVIG therapy and two (18.19%) patients demonstrated liver enzymes within reference ranges (Table 1). None of these patients became symptomatic. Their transaminase levels achieved reference range after 8-10 days of discontinuing the infusion. The outcomes following complications of IVIG were favorable in all patients, and the changes were not clinically relevant.

**Table 1:** Transaminase level at baseline and during intravenous immunoglobulin infusion GBS, Guillain-Barré syndrome; AMAN, acute motor axonal neuropathy; AIDP, acute inflammatory demyelinating polyneuropathy; AMSAN, acute motor sensory axonal neuropathy; U/L, units per liter; AST, aspartate transaminase; ALT, alanine transaminase.

Serial Number	Age (Years)	Sex	Name of Autoimmune Disorder	AST Level (U/L) Baseline	AST Level (U/L) During Treatment	ALT Level (U/L) Baseline	ALT Level (U/L) During Treatment
1	16	Male	GBS (AMSAN)	20	40.25	88	92.5
2	17	Male	GBS (AMSAN)	47	31	35	29
3	25	Female	GBS (AMAN)	24	138.25	31	155.16
4	26	Female	GBS (AIDP)	36	130	24	244
5	31	Male	GBS (AIDP)	30	168.66	45	200.16
6	35	Male	GBS (AIDP)	37	144.66	40	278
7	45	Female	GBS (AMSAN)	30	222	32	169
8	50	Male	GBS (AIDP)	29	57	38	138
9	56	Female	GBS (AIDP)	21	54.5	23	35.0
10	57	Female	GBS (AMAN)	20	149.25	31	159.55
11	45	Male	Myasthenic crisis	36	53	40	179

## Discussion

All patients received IVIG in a dose of 0.4 gm/kg body weight daily in divided doses over five days. After 48-72 hours of administration of IVIG, there was an elevation of liver enzymes in 81.81% (n=9) of patients and reference range liver enzymes in 18.19% patients (n=2). Aspartate transaminase (AST)/alanine transaminase (ALT) reached healthy levels after 5-10 days of discontinuing treatment. Patients who developed transaminitis became normal after discontinuing IVIG infusion. No patients received any hepatatoxic medications during the treatment period. Treatment with IVIG is usually safe; however, substantially adverse effects have also been noticed in the past. All these adverse effects have been reported in retrospective studies or case reports [3,4]. Most reactions were mild and resolved spontaneously. Additionally, previous studies have cited severe reactions also occurring during the treatment course. Leg pain and headache are the most common adverse effects described in 30% of the treatments [4,5]; however, in our study, no such events happened.

Patient age, sex, and underlying disease were not associated with adverse effects of IVIG. Other adverse effects such as elevated liver enzymes, including ALT and AST, were associated with the type of preparation of IVIG used; however, this mechanism has not been elucidated, and another explanation, such as hepatocytic injury via Fas-mediated apoptosis, may be proposed [6]. An earlier study demonstrated that preparations containing sucrose and polyethylene glycol/sorbitol caused elevation of liver enzymes, while no liver enzyme elevation was seen in glycine-containing preparations [5]. All our patients received IVIG containing maltose as a stabilizing agent. We determined that the stabilizing agent in IVIG preparations had a role in the development of transaminitis; however, it was mild and resolved after discontinuation of treatment, and none of the patients became symptomatic, which confirmed the self-limiting adverse effect of IVIG.

## Conclusions

Adverse effects such as transaminitis may be caused by maltose-containing stabilizing agents in IVIG preparation; however, it is a self-limiting phenomenon. Although the incidence of complications with IVIG therapy is rare, one must be careful about monitoring for severe adverse effects. Therefore, before beginning treatment, patients need to be routinely given a complete blood count test, renal function test, and liver function test. Early recognition of complications is of crucial importance in reducing potential harmful effects, and further studies involving a large sample size are required to elucidate the etiopathogenesis of transaminitis.
